# Cultural diversity in beliefs regarding mental illness: comparison of Indonesian Muslims, Indonesian Christians, and Japanese non-religions

**DOI:** 10.3389/fpsyg.2025.1524680

**Published:** 2025-02-28

**Authors:** Shiho Tanaka, Venie Viktoria Rondang Maulina, Theresia Indira Shanti, Medwin Wisnu Prabowo

**Affiliations:** ^1^Department of Psychology, Graduate School of Human Sciences, Sophia University, Tokyo, Japan; ^2^RIKEN Center for Brain Science, Wako, Japan; ^3^International Research Center for Neurointelligence (WPI-IRCN), The University of Tokyo Institutes for Advance Study, Tokyo, Japan; ^4^Department of Psychology, Atma Jaya Catholic University of Indonesia, Jakarta, Indonesia; ^5^Hermina Galaxy Bekasi Hospital, Jakarta, Indonesia

**Keywords:** mental health literacy, Indonesia, Muslim, Japan, depression, cultural diversity, beliefs about mental illness

## Abstract

A majority of Indonesians, approximately 90% of them, are Muslims and have been reported to religiously cope with symptoms of mental illness. This may depend on the degree of recognition of Western medicine and attribution to biological factors; however, this has not been adequately investigated. To avoid clinical bias, it is vital to understand the underlying mechanism. Hence, this study aims to determine the relationship between depression recognition, causal attributions, and coping behaviors among Indonesian Muslims using a quantitative approach. To capture the salient features of Indonesian Muslims, they were compared with Japanese non-religions and Indonesian Christians. We also developed new causal attribution and coping behavior scales that are culturally valid for both Indonesia and Japan. Specifically, in Study 1, we first examined the demographic details of the participants and then developed the scales. In Study 2, we compared Indonesian Muslims, Japanese non-religions, and Indonesian Christians in terms of relationships between recognizing depression, causal attributions, and coping behaviors. Participants were university students around the capital of both countries: 236 Indonesian Muslims (182 females), 493 Japanese non-religions (365 females), and 266 Indonesian Christians (180 females). In Study 1, religiosity was the only demographic characteristic that saliently differed between the 3 groups. Both the causal attribution and coping behavior scales were adequate for use in Study 2. In Study 2, the results of structural equation modeling revealed that the relationships between depression recognition, causal attributions, and coping behaviors differed among Indonesian Muslims, Japanese non-religions, and Indonesian Christians. More specifically, amongst Indonesian Muslims, the level of depression recognition had a marginally significant positive influence on religious attribution, which in turn had a significant positive influence on evil dispelling. However, Japanese non-religions and Indonesian Christians showed no association between the recognition of depression and religious attribution. These results suggests that the three groups may have different beliefs about depression.

## 1 Introduction

### 1.1 Decrease in Japanese population and increase in Indonesian Muslim residents in Japan

While the Japanese population continues to decline, the population of international residents—foreigners living in Japan over the medium-to long term—continues to grow. For instance, the Japanese population in 2020 decreased by 1,783,000 or 1.4% compared to 2015, whereas the population of international residents increased by 835,000 or 43.6% (Komatsu, 2022).

Particularly, the Indonesian population among international residents in Japan has been increasing. The population of international residents as of June 2022 was the 7th largest, with a population of 83,169, and the population growth rate of 39% compared with the previous year was the highest (Ministry of Justice, 2022). As a majority of the Indonesian population, approximately 90%, are Muslims (Pew Research Center, 2019), approximately 10% are Christians, and the remaining are Hindus and Buddhists (Ministry of Foreign Affairs, 2023). The Indonesian residents of Japan can be predominantly Muslims. Therefore, Japan's cultural diversity needs to identify the characteristics of Indonesian Muslims.

### 1.2 Risk of mental health service gap among Indonesian Muslim residents

Indonesian Muslim residents in Japan are more likely to use domestic mental health services. Statistically, anyone is at risk of developing mental health problems. For example, according to surveys in Indonesia and Japan, about one in five people suffer from some form of mental illness during their lifetime (Ministry of Health, Labour and Welfare, 2017; Peltzer and Pengpid, 2018).

However, for international residents, living in different cultures can lead to cross-cultural stress (Berry, 1970; Oberg, 1960), which further increases the risk of developing mental illnesses (Bhugra, 2004). Furthermore, being Muslim may threaten one's mental health. In the US, cultural adjustment issues, discrimination, and social exclusion may cause mental illness among Muslims (Amri and Bemak, 2013). Cultural adjustment issues have been reported among international Muslim students in Japan (Nakano et al., 2015). Also, in Japan, a general intolerance toward religion, based on the World Values Survey (WVS) (Horie, 2018), and a higher negativity toward Islam compared with other religions are reported (Kobayashi, 2019).

Hence, it is not difficult to imagine that this discrimination and social exclusion toward Muslims could be a risk factor for mental health, particularly among Indonesian Muslims in Japan. Therefore, the use of domestic mental health services among Indonesian Muslim residents is highly likely.

Contrary to this need, there is currently no discussion regarding the religious diversity of mental health service users in Japan (Asahara, 2005). One possible explanation for this is that a majority, approximately 80%, of Japanese are non-religions (Institute of Statistical Mathematics, 2013; Ishi, 2007).

However, failure to consider the religious diversity of Japanese mental health service users could contribute to a mental health service gap among Indonesian Muslim residents. For instance, it has been suggested that a poor understanding of and attitude toward Islam among therapists led to treatment dropouts among Muslim clients in Japan (Onishi, 2013). Moreover, therapists' poor understanding of patients or clients' cultural and religious backgrounds can distort their clinical judgments and attitudes owing to clinical bias (Wisch and Mahalik, 1999).

Furthermore, young Indonesian Muslims in their 20s who come to Japan face an increased risk of mental health problems due to working long hours and living alone for the first time. For instance, a report on the study conditions of Indonesian nursing and care worker candidates in Japan highlighted that the stress of living alone and working extended hours for the first without the support of family or dependents could lead to mental health issues such as headaches, diarrhea, nausea, and insomnia (Okushima, 2010).

Therefore, understanding the appropriate responses from Japanese mental health professionals to the cultural differences seen in Indonesian Muslim clients or patients is important to avoid a therapeutic rupture or disadvantage.

### 1.3 Uniqueness and mechanisms of coping behavior among Indonesian Muslims

Mental Health Literacy (MHL) is defined as “knowledge and beliefs about mental disorders which aid their recognition, management or prevention” (Jorm et al., 1997). The definition has changed somewhat over time (Kutcher et al., 2016). According to Jorm (2012), MHL is an inclusive concept encompassing as (a) the public's knowledge of how to prevent mental disorders, (b) recognition of when a disorder is developing, (c) knowledge of help-seeking options and treatments available, (d) knowledge of effective self-help strategies for milder problems, (e) first aid skills to support others affected by mental health problems. This concept includes recognizing the symptoms of mental illness (e.g., the degree to which symptoms of depression are recognized as the illness “depression”), causal attribution (attribution of reasons for the symptoms of a mental illness), and recognition of coping behaviors (recognition of behaviors in response to symptoms of a mental illness). Coping behavior includes seeking help from mental health professionals.

When Indonesian Muslims have mental health problems, they are observed to cope differently instead of seeking help from mental health professionals. These include praying, going to the mosque, joining religious activities, and receiving religious or traditional treatment (Brooks et al., 2022a). It is also reported that Indonesian Muslims prefer to solve mental health problems with religious or family resources rather than seeking help from mental health professionals (Syafitri, 2022). This way, Indonesian Muslims actively use religious coping toward mental illness.

However, religious coping strategies are uncommon in Japan. First, a survey conducted in Japan found no religious coping mechanisms for mental illnesses (Koenig et al., 2012). Additionally, a study comparing MHL in Japan and Australia (Jorm et al., 2005) found some religious coping, while the number of respondents who said that consultation with a religious leader for depression was helpful was lower in Japan (13.6%) than in Australia (45.3%), where nearly half of the respondents said that consultation with a religious leader was helpful. Conversely, while 8.1% of the respondents in Australia said that consulting a religious practitioner was harmful, three times as many respondents in Japan (24.2%), or one-fourth the number of Japanese said that consulting a religious practitioner was harmful. This finding suggests that religious coping is viewed negatively in Japan. In a more recent study, only 2% of all respondents in Japan reported that having spiritual support, such as religion, was effective in preventing mental illness (Kawabata et al., 2018). This suggests that religious coping with mental illness is uncommon in Japan. Therefore, it is therapeutically important for Japanese mental health professionals to develop an appropriate understanding of the religious coping strategies used by Indonesian Muslims.

According to previous research, two factors are related to such religious coping among Muslims: causal attribution and appropriate Western medical recognition of psychiatric symptoms, both of which are included in the MHL. Some examples of causal attribution toward mental illness related to religious coping are a weak Imaan; things that enhance faith, atonement, or punishment for sin with trials from God; God's will or destiny; black magic; Jinn or devil possession (Wahyuni et al., 2017). Another study reported that supernatural phenomena, such as supernatural powers or ghosts, are also believed to be attributed to mental illness, and these beliefs lead people to seek help from traditional or religious healers (Marthoenis et al., 2016).

Another factor influencing religious coping is the recognition of mental illness symptoms. Symptom recognition refers to the recognition or understanding of mental illness based on Western medicine, such as recognizing depression symptoms as depression, as defined by the Diagnostic and Statistical Mannual of Mental Disorders (DSM) or International Statistical Classification of Diseases and Related Health Problems (ICD). In studies in the field of MHL, the degree of recognition of mental illness was found to influence seeking help from mental health professionals (Yap et al., 2015; Gulliver et al., 2012). However, it has been suggested that the degree of mental illness recognition is relatively low in Indonesia (Brooks et al., 2022a).

In previous MHL studies, religious coping behavior, supernatural causal attribution, and a lack of recognition of mental illness that differ from modern Western medicine were viewed as a lack of MHL, which may hinder mental health service utilization (Jorm, 2012). Religious coping behaviors may be chosen as poor recognition of illness leads to supernatural causal attribution, which in turn leads to religious coping (Widayanti et al., 2020). All of these religious recognition of mental illness and copig behavioral choices were sometimes treated as if they were inaccurate MHLs requiring correction.

However, as far as the authors know, the relationship of the two factors—causal attribution and recognition of mental illness—with religious coping has not yet been examined. Therefore, based on the findings to date, it is uncertain whether causal attributions and coping behaviors that differ from modern Western medicine can be viewed as a lack of MHL. In fact, it is reported that recently in Indonesia, more than 90% of mental health professionals who have appropriate recognition of mental illness and Western medical causal attribution also support the benefits of religious coping, such as seeking help from religious professionals or joining religious activities for depression (Praharso et al., 2020).

Hence, it is crucial to investigate the relationship between the recognition of mental illness, causal attribution, and coping behavior to examine whether a poor recognition of mental illness influences causal attribution, which in turn promotes religious coping, or whether there are other mechanisms.

### 1.4 Models examined in this study

This study focused on depression and examined the relationships among the recognition of depressive symptoms (the degree to which symptoms of depression are recognized as a disease called depression), causal attribution (attribution of reasons for symptoms of depression), and coping behavior (behavior for symptoms of depression) for three particular reasons. First, Jorm et al. (1997) reported that previous MHL studies often used depressive symptoms, making them highly comparable. Second, the degree of depression symptom recognition in Indonesia and Japan is almost the same, which is about 50%−60% (Amaliah et al., 2022; Schlemper, 2019). Therefore, these two populations were considered appropriate for comparison. Third, both countries have a high prevalence of depression. In Japan, 1 in 15 people will suffer from depression at least once in their lives, indicating a high lifetime prevalence (Kawakami, 2006). In Indonesia, 21.8% of the population exhibits symptoms of depression (Peltzer and Pengpid, 2018). Hence, providing appropriate support for those affected by depression is an urgent issue in both countries.

In addition, the model examined in this study is based on that of Altweck et al. (2015). They also made cross-cultural comparisons on the model, and the variables we use in the model are not the same but similar. Hence, considering our study's purpose, we deemed it appropriate to use their model. Therefore, we examined a model in which recognition of depression influences causal attribution, which in turn influences coping behavior, with recognition of depression as the independent variable and causal attribution and coping behavior as the dependent variables.

However, in their study, cross-cultural comparisons were conducted to identify the difference between individualism and collectivism but not between religious cultures. Therefore, it is important to compare the Indonesian and Japanese non-religion cultures in this study. Hence, it is crucial in this study to understand the key traits of Indonesian Muslims, the majority in Indonesia. For this purpose, we compared Indonesian Muslims with Japanese non-religions, the majority in Japan, and Indonesian Christians, the second largest group in Indonesia. There are other religious populations, such as Hindus and Buddhists, in Indonesia, but as mentioned earlier, their populations are relatively small. This study requires a large sample size to examine a complex model. While it is important to investigate Hindus and Buddhists for our purposes, we determined that Christians were appropriate for this study.

Furthermore, to measure one of the variables in the model, depression recognition, we used the Depression Recognition Scale, which can be used for Indonesian and Japanese populations (Tanaka and Hisata, 2022; Tanaka et al., 2024). However, to the best of our knowledge, there is no appropriate scale to measure causal attribution and coping behavior among Indonesian and Japanese populations. Therefore, we developed these scales for the present study. For details on the indicators and items of each variable, please refer to the Sections 3 and 4.

## 2 Purpose

This study aimed to determine the relationship between depression recognition, causal attributions, and coping behaviors using a quantitative approach. Japanese non-religions and Indonesian Christians were compared to capture the salient features of Indonesian Muslims. We also developed new causal attribution and coping behavior scales appropriate for Indonesian and Japanese populations. Specifically, in Study 1, we first examined the background and demographic details of the participants and then developed the scales. In Study 2, we compared the relationships between the three variables among Indonesian Muslims, Japanese non-religions, and Indonesian Christians.

## 3 Methods

### 3.1 Participants

The degree of acculturation into the host society influences attitudes toward aid resources (Atkinson and Gim, 1989). Therefore, this study focused on a subject that is expected to have significant cultural conflict with Japan: Indonesian Muslims who have never lived in Japan and are considered to have little influence from non-Indonesian cultures.

Studies have shown that 75% of lifetime cases of psychiatric disorders occur by the mid-twenties (Kessler et al., 2005). In other words, most mental illnesses develop by the approximate age of college students. In addition, the 12-month prevalence rate for depression among college students has been found to be 18.5 % (Auerbach et al., 2018). Also, in Japan, 20.7% of first-year college students had a major depressive episode within 1 year (Tomoda et al., 2000). Approximately one in four to five college students suffers from depression, highlighting the need for immediate countermeasures.

Nevertheless, college students have lower intentions to seek help from mental health professionals than from informal subjects, such as family and friends (Nagai, 2010). In addition, there are concerns about the long-term adverse effects of delayed treatment in college students, not only immediate adverse effects such as disruption of academic performance, but also the risk of recurrence in adulthood (American College Health Association, 2009). Furthermore, coping behavior styles, such as help-seeking behavior, formed in the age group that generally corresponds to college years, persist throughout life (Schonert-Reichl, 2003). Providing appropriate support to affected individuals by college age can significantly improve their prognosis in the following years.

Furthermore, there were concerns about the influence of educational background on MHL investigated in this study. Therefore, in addition to the reasons above, to control for this educational effect, we included current college students in both countries.

The participants were undergraduate and graduate students attending universities in Indonesia and Japan's metropolitan areas around Jakarta and Tokyo, the capital cities of both countries. This was done to eliminate the effects of regional disparities.

This study examined the relationships among depression recognition, causal attribution, and coping behaviors. Therefore, efforts were made to include students from a various majors so that the degree of knowledge and understanding of mental illness varied among participants; for example, to avoid including only students who were familiar with mental illness, psychiatry, and clinical psychology. As a result, we included students from 24 academic disciplines in Japan and 28 in Indonesia, including disciplines such as linguistics, literature, biology, economics, philosophy, physics, psychology, and pharmacy.

Moreover, as our purpose was a cultural comparison, only students who met the following criteria were included in the analysis: (a) their country of origin must be either Indonesia or Japan; (b) their native language must be either Indonesian or Japanese; (c) they must have stayed abroad for < 1 month; and (d) their parents must be from the same country as the participant. Additionally, we defined Indonesian participants who self-reported their religion of faith as Islam as Indonesian Muslims, and as Christianity as Indonesian Christians, and Japanese participants who answered “no religion” as Japanese non-religions.

Furthermore, when examining a model using Structural Equation Modeling (SEM), a sample with five times the number of coefficients was sought (Bentler and Chou, 1987). However, because the appropriate sample size for the model can be determined from many different angles, there is no clear answer at this point, and we need as large a sample as possible (Kawashi, 2009). In this study, we estimated that a large number of coefficients would be required to examine a complex model. Therefore, recruiting a larger sample of at least 200 individuals in each group was necessary.

Consequently, 995 participants were included in the analysis: 236 Indonesian Muslims (182 women, 0 other), 493 Japanese non-religions (365 women, 3 others), and 266 Indonesian Christians (180 women, 4 others).

### 3.2 Procedure

Data were collected from November 2021 to March 2022 via online surveys. The URL for the online survey was disseminated before or after classes in Japan, and in Indonesia, a mailing list was also used. No compensation was offered to participants.

### 3.3 Translation

In addition to the Depression Recognition Scale (Tanaka et al., 2024), we had to translate the Japanese questionnaire into Indonesian. The back-translation method was used for the translation. A professional translator translated the original Japanese version (a) into Indonesian (a). Another professional translator translated the Indonesian version (a) into Japanese (b). A Japanese psychologist and a Japanese graduate student majoring in psychology compared Japanese versions (a) and (b) to check if the meaning of the content was similar. This procedure was repeated until all items of Japanese (a) and (b) became equal. Finally, an Indonesian psychologist slightly modified the Indonesian content to make it appropriate for psychological surveys in Indonesia.

### 3.4 Statistical analysis

IBM SPSS 27/28 and Amos 27/28 were used for data analysis. In Study 1, first, basic statistics were calculated for the demographic of participants, and whether significant group differences were found for each demographic was examined using the χ^2^ test or one-way analysis of variance (ANOVA).

Second, the following analyses were conducted to develop the new causal attribution scale. Developing an entirely common scale suitable for different cultures is challenging due to linguistic and cultural differences. Therefore, the aim of developing a scale in this study was to create an index that would be useful in examining how relationships among the targeted variables differed among the three groups that are the focus of this study, rather than developing a highly universal scale that can be used in all cases. As the purpose of this study was to compare three groups—Indonesian Muslims, Japanese non-religions, and Indonesian Christians—it was necessary to establish a common factor structure for all groups. Therefore, all the samples were examined for common factor structures in an exploratory factor analysis. We determined the validity of the factor structure obtained for the entire sample, which was adaptable for each of the three groups, using the goodness-of-fit index of the model during the simultaneous multigroup analysis.

If the goodness-of-fit index of the model is adequate (configural invariance), that is, the model is guaranteed to be similar among the groups being compared (Toyoda, 2007), it can be inferred that the factor structure derived from the entire sample is adaptable for each of the three groups. The results of this validity are discussed in detail when examining the model fit of the simultaneous multi-group analysis in Study 2. This was also the case for the Coping Behavior Scale.

Additionally, from a reliability perspective, if the results of the factor analysis raised concerns about attenuation due to alpha values or other factors, these issues were addressed in Study 2 by using latent variables to correct for attenuation (see Section 4.1.2 for details).

Therefore, in selecting the questionnaire items, we referred to previous studies (e.g., Schlemper, 2019; Praharso et al., 2020; Tanaka and Hisata, 2021) ensuring they reflected the cultural background of two countries, simultaneously maintaining equivalence. Consequently, 17 tentative items were selected.

Given the widespread use of vignette-based method to assess belief about mental illness globally (Jorm, 2019), we opted for this approach. Participants read the vignette (see Section 3.6.8. Depression recognition), and were then asked, “If you were in this condition, why do you think this happened to you?” and were asked to respond to each of these 17 items on a 4-point scale, ranging from “1. Unlikely” to “4. Very likely,” for each possibility. The participants were randomly presented with 17 items.

Third, the following analyses were conducted to develop a new coping behavior scale: In selecting the questionnaire items, we referred to previous studies (e.g., Nakamura and Hisata, 2008; Praharso et al., 2020; Tanaka and Hisata, 2021) to ensure that they reflected the cultural background of both countries and maintained equivalence. Consequently, 26 items were selected. The scale was presented, and respondents responded to in the same manner as the causal attribution scale.

In Study 2, we compared Indonesian Muslims, Japanese non-religions, and Indonesian Christians to examine the relationship between depression recognition, causal attribution, and coping behavior.

**Figure 1:**
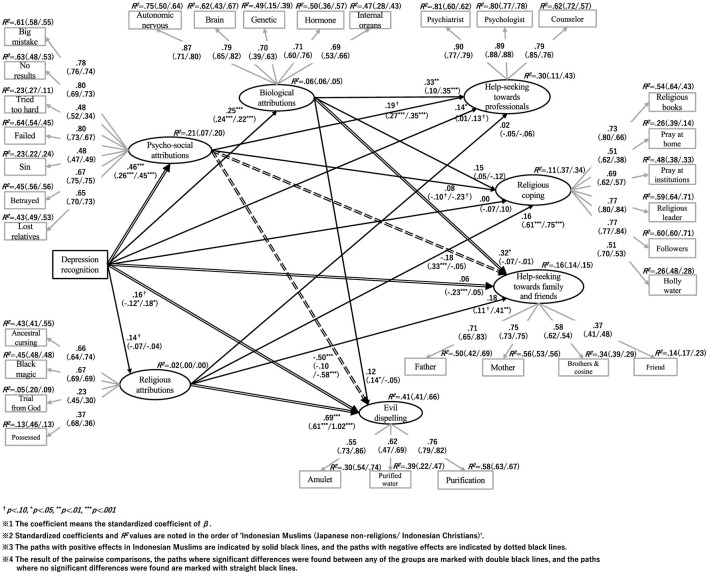
Group comparisons in the relationship between depression recognition, causal attributions, and coping behaviors.

A model was created with depression recognition as the observed variable and the subscales of causal attribution and coping behavior, for example, psychosocial attribution and help-seeking from (mental health) professionals, as latent variables; the difference in association between these variables among the three groups was examined using simultaneous multigroup analysis in SEM. The structure of the model under consideration was one in which depression recognition influenced the subscales of causal attributions and coping behavior subscales, and the causal attributions subscale further influenced the coping behaviors subscale (Figure 1).

### 3.5 Ethical considerations

The study was approved by the Sophia University's “Human study” ethics committee [No. 2021-22 (87, 145), 2021-76 (127, 146)], and by the ethics committee of Atma Jaya Catholic University of Indonesia (No. 0030D/III/LPPM-PM.10.05/10/2021). As an ethical consideration, consent to participate in the study was obtained before participants began answering the questionnaire. Specifically, we explained on the top screen of the online survey that the responses would be collected anonymously, that participation in the study was voluntary, and that there would be no disadvantages to their grades or other evaluations if they refused to participate or stopped answering the questionnaire midway through the survey. Only those who clicked the “Agree” button were considered to have agreed to participate in the study.

### 3.6 Measurements

#### 3.6.1 Age, gender, nationality, and native language

Participants responded numerically for age; chose either female, male, or other for gender; chose Indonesia or Japan for nationality; chose Indonesian, Japanese, or other for native language. When participants chose “other” as their answer, they were asked to provide further details.

#### 3.6.2 Religion

Participants chose either Buddhism, Shintoism, Christianity, Islam, non-religions, or other in the Japanese survey; they chose Islam, Christianity (Catholic, Protestant), Buddhism, Hindu, or other in the Indonesian survey. When participants chose “other” as their answer, they were asked to provide further details.

#### 3.6.3 History of psychiatric consultation and counseling

Participants chose “yes” or “no” for whether they themselves or their families had consulted with psychiatrists or counselors to indicate their or their family's history of psychiatric consultation and counseling.

#### 3.6.4 History of psychiatric study

Participants chose one of the four options, ranging from “1. None” to “4. Have studied independently by all means” to indicate their previous experience with psychiatric studies.

#### 3.6.5 Economic conditions

Participants chose one from five alternatives, ranging from “1. High” to “5. Low” to indicate the family's economic situation in their respective countries. As smaller numbers indicate better economic conditions, the analytical results are presented with an inversion.

#### 3.6.6 Religiosity

We measured internal religiosity according to the Japanese version of the Duke University Religion Index (DUREL; Gonzales et al., 2015) using an original English scale developed by Koenig and Bussing (2010). It consisted of 3 items such as “I have felt a holy presence (God's presence) in my life.” Participants were asked to rate the degree to which they believed each condition applied best to them—ranging from “1. Not applicable at all” to “5. Complementarily applicable”—regardless of their religious affiliation.

Cronbach's alpha coefficients (α) were 0.94 (total sample), 0.79 (Indonesian Muslims), 0.61 (Japanese non-religions), and 0.88 (Indonesian Christians). The Cronbach's alpha for the Japanese non-religions population was lower. However, as the purpose of this variable was to use it for establishing the validity of the subscales of causal attribution and coping behavior for the total sample, we included this value in later analyses because the value of the total sample was high.

#### 3.6.7 Self-stigma of seeking help of mental health professionals

We used the unidimensional 10-item Japanese version of Self-Stigma of Seeking Help (SSOSH; Ina and Morita, 2015), which was originally developed in English (Vogel et al., 2006). We modified the expression “therapist” in the English version and “counselor” in the Japanese version to “psychiatrist and psychologist” to clarify the expression for Indonesian and Japanese circumstances. Participants were asked to choose one of five levels, which applied to themselves to questions such as, “If I went to a psychiatrist or psychologist for advice, I would feel helpless.” A total of 10 questions were presented in random order to the participants.

Cronbach's alpha coefficients (α) were 0.69 (total sample), 0.50 (Indonesian Muslims), 0.80 (Japanese non-religions), and 0.56 (Indonesian Christians). Indonesian Muslims and Christians had low Cronbach's alpha values. However, we included this variable in a later analysis for the same reason as the “religiosity” variable mentioned earlier.

#### 3.6.8 Depression recognition

We used the Depression Recognition Scales, which have been developed for both Japanese and Indonesian populations (Tanaka and Hisata, 2022; Tanaka et al., 2024). Participants were first asked to read the vignette describing depression symptoms such as “You have been feeling unusually sad and miserable for the last 1 month. You are always tired and cannot sleep well. You have no appetite and lost weight. You are restless and irritable and have no motivation to do anything. You are unable to concentrate on schoolwork and delay decisions that need to be made, and even daily routine activities such as brushing teeth and bathing feel like a burden.”

They were then asked to hypothetically imagine themselves in this state and to respond to a total of 10 possible conditions, which included five mental illness items [depression, schizophrenia, panic disorder, post-traumatic stress disorder (PTSD), and anorexia] and five physical illness items (asthma, sleep apnea syndrome, hyperthyroidism, diabetes, and gastric ulcer), using a 4-point scale: “1. Unlikely,” “2. Not very likely,” “3. Highly likely,” and “4. Very high likely.” The 10 items were randomly presented to participants, and only the five mental illness items were scored based on Tanaka and Hisata (2022). More specifically, first, total scores were calculated using the mean of 4 items of the mental illness identification scale, which excluded the depression-related item. Finally, using the mean score of the mental illness identification scale, the total depression scores were calculated as “1” for those who selected 2 or fewer for the depression item, “2” for those who selected 3 or more for the depression item and whose total score on the mental illness identification scale was above average, and “3” for those who selected 3 or more for the depression item and whose total score on the mental illness identification scale was above average. The higher the score, the higher the likelihood of depression, and the less likely were the other four mental disorders.

Cronbach's alpha coefficients (α) were 0.73 (total sample), 0.70 (Indonesian Muslims), 0.77 (Japanese non-religions), and 0.72 (Indonesian Christians). As all groups showed high internal consistency, we included this variable in the subsequent analysis.

## 4 Results

### 4.1 Study 1. Basic statistics of valuables and scale development

#### 4.1.1 Demographic of participants for each of the three groups

To see if there was any considerable demographic difference, we calculated basic statistics for the age of participants, gender, history of psychiatric consultation by themselves, history of psychiatric consultation by their family, history of psychologist counseling by themselves, history of psychologist counseling by their family, history of psychiatric studies, economic status, religiosity, and self-stigma of help-seeking from mental health professionals. The results are summarized in Table 1. Depending on the characteristics of the variables, we conducted χ^2^ test or one-way ANOVA to compare the three groups. Significant differences were observed for some variables. However, for variables with significant differences, there were no big effect sizes found: age (η^2^ = 0.05), history of psychologist counseling by themselves (*V* = 0.36), history of psychologist counseling by their family (*V* = 0.17), history of psychiatric studies (η^2^ = 0.03), economic status (η^2^ = 0.03). For religiosity (η^2^ = 0.76), a relatively large effect size was observed, but the results were considered reasonable for the study's purpose.

**Table 1:** Basic statistics on participant demographics.

		**Indonesian Muslims**		**Japanese non-religions**		**Indonesian Christians**		
**Variables**	**Classification**	**Frequency (%)**	**Mean (*SD*)**	**Frequency (%)**	**Mean *(SD)***	**Frequency (%)**	**Mean (*SD*)**	**Statistic**
Age			20.70 (2.62)		19.65 (1.89)		20.52 (2.17)	*F*_(2, 115.04)_ = 24.73^***^, η^2^ = 0.05, 95%CI = 0.02–0.08
Gender	Female	182 (77.1)		365 (74)		180 (67.7)		$\chi_{\left(4\right)}^{2}$ = 9.10, *n.s*., *V* = 0.07
	Male	54 (22.9)		125 (25.4)		82 (30.8)		
	Other	0 (0)		3 (0.6)		4 (1.5)		
Psychiatric consultation history by themselves	Yes	19 (8.1)		54 (11)		31 (11.7)		χ^2^_(2)_ = 2.00, *n.s., V* = 0.05
	No	217 (91.9)		439 (89)		235 (88.3)		
Psychiatric consultation history by their family	Yes	69 (29.2)		128 (26)		80 (30.1)		χ^2^_(2)_ = 1.76, *n.s., V* = 0.04
	No	167 (70.8)		365 (74)		186 (69.9)		
Psychologist counseling history by themselves	Yes	102 (43.2)		132 (26.8)		186 (69.9)		χ^2^_(2)_ = 132.01^***^, *V* = 0.36
	No	134 (56.8)		361 (73.2)		80 (30)		
Psychologist counseling history by their family	Yes	100 (42.4)		142 (28.8)		127 (47.7)		χ^2^_(2)_ = 30.27^***^, *V* = 0.17
	No	136 (57.6)		351 (71.2)		139 (52.3)		
History of psychiatric studies			2.12 (0.87)		1.78 (0.93)		1.98 (0.84)	*F*_(2, 10.11)_ = 12.66^***^, η^2^ = 0.03, 95%CI = 0.01–0.05
Economic status			3.12 (0.74)		3.45 (0.83)		3.26 (0.66)	*F*_(2, 9.45)_ = 16.14^***^, η^2^ = 0.03, 95%CI = 0.01–0.06
Religiosity			4.41 (0.76)		1.52 (0.61)		4.04 (0.96)	*F*_(2, 909.99)_ = 1,589.64^***^, η^2^ = 0.76, 95%CI = 0.74–0.78
Self-stigma of help-seeking			2.42 (0.50)		2.50 (0.67)		2.43 (0.53)	*F*_(2, 0.76)_ = 2.15, *n.s*., η^2^ = 0.00, 95%CI = 0.00–0.02

^*^p < 0.05, ^**^p < 0.01, ^***^p < 0.001.

#### 4.1.2 Causal attribution scale

As described in the Statistical Analysis section of the Methods, 17 items were used.

First, a graduation student and a psychologist examined the trends in ceiling and floor effects based on the distribution of tentative item scores and mean ± SD of the 17 tentative items and examined the items considering the aim of the study. Consequently, all the 17 tentative items were included for further analysis.

Next, an exploratory factor analysis (EFA) was performed on the 17 items using the maximum likelihood method and ProMax rotation. A three-factor structure was considered reasonable with reference to the eigenvalues and scree plots. The EFA was conducted again based on these factors. Using the factor loading of 0.40 as a guide, double-loading items were removed, which resulted in three factors and 16 items (Table 2).

**Table 2:** Pattern matrix of causal attributions.

	**F1**	**F2**	**F3**	**Communality**
No matter how hard I try, I can't get results	**0.784**	−0.047	0.031	0.587
Big mistake	**0.776**	0.023	−0.008	0.619
Failed	**0.735**	−0.067	0.104	0.524
Betrayed by someone you trusted	**0.694**	0.047	0.049	0.527
Lost someone very close to me	**0.597**	0.198	−0.047	0.511
I have sinned	**0.458**	−0.049	0.310	0.320
Tried too hard	**0.414**	0.171	−0.126	0.276
Abnormality of the autonomic nervous system	0.168	**0.713**	−0.248	0.715
Brain anomalies	0.069	**0.707**	0.090	0.561
Hormonal abnormalities	0.155	**0.637**	−0.120	0.542
Abnormality of internal organs	−0.056	**0.637**	0.127	0.384
Genetic problems	−0.077	**0.567**	0.186	0.310
Possessed by something	0.131	−0.073	**0.606**	0.401
Trial from God	0.134	−0.250	**0.566**	0.391
Ancestral cursing	−0.124	0.379	**0.566**	0.406
Be under black magic	−0.091	0.332	**0.554**	0.376
Factor correlations F1	-	0.503	0.121	
F2		-	−0.02	

Bold letters mean that the factor loadings were 0.40 or higher.

The first factor was named psychosocial attribution because it included seven items representing the burden due to psychological or interpersonal involvement. The second factor, named biological attribution, consisted of five items representing physical issues. The third factor, named religious attribution, consisted of four items representing supernatural issues or religious matters.

Cronbach's alpha using all samples for the factors were as follows: α = 0.84 for psychosocial attribution, α = 0.81 for biological attribution, and α = 0.61 for religious attribution. In addition, we calculated the Cronbach's alpha for each of the three groups (Table 3). Psychosocial attribution and biological attribution had sufficient internal consistency (α = 0.72–0.86) in both factors for all samples and each of the three groups. However, the “religious attribution” factor had a slightly lower α, especially in Indonesian Muslims and Christians, with 0.44 and 0.52, respectively.

**Table 3:** Basic statistics on causal attributions.

	**Indonesian Muslims**	**Japanese non-religions**	**Indonesian Christians**
**Factor names**	**Mean (*SD*) (α)**	**Mean (*SD*) (α)**	**Mean (*SD*) (α)**
Psycho-social attributions	2.57 (0.77) (0.84)	3.02 (0.62) (0.84)	2.74 (0.73) (0.83)
Biological attributions	1.74 (0.73) (0.86)	2.44 (0.59) (0.72)	2.00 (0.76) (0.85)
Religious attributions	1.83 (0.49) (0.44)	1.28 (0.41) (0.68)	1.80 (0.54) (0.52)

When variables with low α values are used in the SEM analysis as observed variables, they are affected by measurement error; that is, an attenuation problem arises, which causes the correlations between variables to be lower than they are (Kano, 1998; Toyoda, 2003). However, in Study 2, to solve this problem, all variables, including religious attribution, were entered into the analysis as latent variables rather than observed variables, thereby correcting for this attenuation (Kano, 1998; Toyoda, 2003). Thus, the results of the SEM analysis were computed decoupled from the effects caused by measurement error.

To examine the concomitant validity of religious attribution, the correlation coefficient between religious attribution and religiosity was calculated for all samples. The results showed a significant moderate positive correlation of *r* = 0.47 (*p* < 0.001), establishing the factor's validity.

Given the above, the three-factor structure—psychosocial, biological, and religious attribution—was appropriate for the construction of causal attributions for all samples.

The descriptive statistics for the three factors of causal attribution are shown in Table 3.

#### 4.1.3 Coping behavior scale

As described in the Statistical Analysis section of the Methods, 26 items were used.

First, a graduation student and a psychologist examined the trends in ceiling and floor effects based on the distribution of tentative item scores and mean ± SD of the 26 tentative items and examined the items considering the aim of the study. Consequently, 18 items were included as tentative items.

Then, an EFA was performed on the 18 items using the maximum likelihood method and ProMax rotation. A four-factor structure was considered reasonable, considering the eigenvalues and scree plots. An EFA was conducted again based on these factors. Using a factor loading of 0.40 as a guide, double-loading items were removed, and consequently, four factors with 16 items were extracted (Table 4).

**Table 4:** Pattern matrix of coping behavior.

	**F1**	**F2**	**F3**	**F4**	**Communality**
Read books related to religion	**0.912**	−0.036	−0.003	−0.150	0.761
Pray at home	**0.814**	−0.091	−0.065	−0.153	0.595
Consult with followers of the same religion	**0.791**	0.034	0.063	0.015	0.665
Consult with a religious leader	**0.740**	0.095	0.016	0.100	0.647
Go to shrines/temples/mosques/churches, etc. to pray	**0.684**	0.015	0.016	0.087	0.522
Drink holy water	**0.486**	0.000	−0.035	0.285	0.394
Consult with a psychologist	0.073	**0.902**	−0.067	−0.045	0.781
Consult with a psychiatrist	−0.081	**0.801**	0.012	0.057	0.653
Consult with a counselor	−0.004	**0.789**	0.070	−0.047	0.658
Consult with a mother	−0.047	−0.020	**0.763**	0.026	0.575
Consult with a father	0.073	0.015	**0.694**	−0.022	0.501
Consult with brothers and cousin	0.043	−0.031	**0.602**	−0.013	0.352
Consult with a friend	−0.068	0.048	**0.437**	0.003	0.206
Have a purification performed	0.011	−0.035	−0.003	**0.816**	0.661
Buy an amulet or talisman	−0.091	−0.002	0.014	**0.768**	0.562
Use the purified water (drink it or bathe in it)	0.294	−0.002	−0.027	**0.416**	0.328
Factor correlations F1	-	0.169	0.160	0.318	
F2		-	0.422	0.191	
F3			-	0.366	

Bold letters mean that the factor loadings were 0.40 or higher.

The first factor was named religious coping because it included six items representing coping behavior in a religious way. The second factor was termed help-seeking from professionals, as it consisted of three items related to seeking help from mental health professionals. The third factor was named help-seeking from family and friends because it consisted of four items related to seeking help from family and friends.

Additionally, the fourth factor consisted of 3 items, “buy an amulet or talisman,” “have a purification,” and “use the purified water (drink it or bathe in it).” In Japan, it is customary to exorcize bad luck by offering good luck charms, *omamori* (amulet), and prayers or exorcisms to ward off bad luck. In addition, in Islamic culture, sometimes the cause of mental illness is attributed to supernatural factors, such as *jinn*, the evil eye (*nazar)*, or punishment or ordeal from Allah, God (Al-Krenawi and Graham, 2011). For the envy of people, called the evil eye, there is an amulet called the Hand of Fatima that exorcizes it (Sayed, 2016). There is also a ceremony to exorcize bad spirits called jinn (Saito, 2017). Furthermore, in Islam, the Qur'an is used for healing, where people drink water while the Qur'an is recited, or water with a verse from the Qur'an dissolved in it is used (Kruk, 2005). Even in Christianity, hanging a cross around one's neck protects one from evil spirits (Samaan, 2010). Christianity, especially Catholicism, recognizes exorcism, and the International Association of Exorcists, formed by priests who practice exorcism, is officially recognized by the Vatican (Roman Curia; Christian Today, 2014; Independent, 2014). As we were able to determine that the items consisted of content representing the payment of bad things from a religious perspective, we named the factor evil dispelling.

Cronbach's alpha for all samples were as follows: α = 0.88 for religious coping, α = 0.87 for help-seeking from professionals, α = 0.72 for help-seeking from family and friends, and α = 0.70 for evil dispelling. In addition, we calculated the Cronbach's alpha for each of the three groups (Table 5). As a result, religious coping, help-seeking from professionals, and help-seeking from family and friends had sufficient internal consistency (α = 0.69–0.89) for all samples and each of the three groups. However, a slightly lower α was observed for Indonesian Muslims (α = 0.64) in evil dispelling.

**Table 5:** Basic statistics on coping behaviors.

	**Indonesian Muslims**	**Japanese non-religions**	**Indonesian Christians**
**Factor names**	**Mean (*SD*) (α)**	**Mean (*SD*) (α)**	**Mean (*SD*) (α)**
Religious coping	2.14 (0.70) (0.83)	1.15 (0.33) (0.83)	2.03 (0.68) (0.81)
Help-seeking toward professionals	2.43 (1.07) (0.89)	2.68 (0.95) (0.87)	2.78 (0.99) (0.85)
Help-seeking toward family and friends	2.17 (0.73) (0.70)	2.61 (0.72) (0.69)	2.40 (0.76) (0.75)
Evil dispelling	1.26 (0.43) (0.64)	1.33 (0.52) (0.77)	1.31 (0.51) (0.81)

As with the causal attribution scale, to address the issue of attenuation due to a lower α value, in Study 2, all coping behavior variables, including evil dispelling, are entered into the SEM as latent variables for analysis.

To examine the concomitant validity of help-seeking from professionals, the correlation coefficient between help-seeking from mental health professionals and the self-stigma of seeking help from mental health professionals was calculated for all samples. The result showed a significant moderate negative correlation of *r* = −0.23 (*p* < 0.001), indicating adequate validity for the factor.

Then, the concomitant validity of religious coping was examined via the correlation between religious coping and religiosity for all samples. The results showed a significant moderate positive correlation of *r* = 0.71 (*p* < 0.001), indicating adequate validity.

Similarly, the concomitant validity of evil dispelling was examined. The result showed no significant correlation (*r* = 0.03; *p* = 0.368), indicating no relationship between the degree of religiosity and evil dispelling.

However, as mentioned earlier, the items included in the evil dispelling were different from the items included in religious coping, such as “praying” and “reading books related to religion (Quran/Bible/Scripture/Hadith, etc.),” which are not necessarily part of one's daily religiosity. Therefore, despite a lack of association with religiosity, it could not be considered entirely inappropriate, and there was no problem with using this factor in Study 2.

Given the above, the four factors of religious coping, help-seeking from professionals, help-seeking from family and friends, and evil dispelling were considered appropriate for constructing the coping behavior scale for all samples.

The descriptive statistics for the four factors of coping behavior are shown in Table 5.

#### 4.1.4 Descriptive statistics of depression recognition

Sufficient internal consistency was also confirmed. The descriptive statistics for depression recognition for each group are presented in Table 6.

**Table 6:** Basic statistic of depression recognition.

	**Indonesian Muslims**	**Japanese non-religions**	**Indonesian Christians**
	**Mean (*SD*) (α)**	**Mean (*SD*) (α)**	**Mean (*SD*) (α)**
Depression recognition	1.96 (0.95) (0.70)	2.27 (0.75) (0.77)	2.12 (0.91) (0.72)

#### 4.1.5 Correlation among eight variables used in Study 2

Finally, the correlation coefficients (*r*) among the eight variables used in structural equation modeling were calculated separately for the three groups. The result is shown in Table 7.

**Table 7:** Correlation among variables in three groups.

	**1**	**2**	**3**	**4**	**5**	**6**	**7**	**8**
1. Depresion recognition	-	0.416^**^/0.408^**^	0.249^**^/0.199^**^	0.156^*^/0.048	0.296^**^/0.329^**^	0.094/−0.086	0.086 /0.039	0.048/−0.128^*^
2. Psycho-social attributions	0.237^**^	-	0.491^**^/0.493^**^	0.589^**^/0.530^**^	0.407^**^/0.490^**^	0.251^**^/0.113	0.160^*^/0.215^**^	0.030/0.037
3.Biological attributions	0.175^**^	0.381^**^	-	0.420^**^/0.438^**^	0.430^**^/0.468^**^	0.245^**^/0.163^**^	0.292^**^/0.223^**^	0.273^**^/0.271^**^
4. Religious attributions	−0.058	0.177^**^	0.104^*^	-	0.211^**^/0.256^**^	0.308^**^/0.369^**^	0.223^**^/0.309^**^	0.197^**^/0.420^**^
5. Help-seeking toward professionals	0.104^*^	0.284^**^	0.190^**^	−0.008	-	0.387^**^/0.218^**^	0.251^**^/0.319^**^	0.216^**^/0.137^*^
6. Religious coping	−0.138^**^	0.029	0.067	0.478^**^	0.060	-	0.463^**^/0.475^**^	0.411^**^/0.486^**^
7. Help-seeking toward family and friends	−0.138^**^	0.218^**^	0.064	0.125^**^	0.383^**^	0.212^**^	-	0.321^**^/0.288^**^
8. Evil dispelling	−0.134^**^	0.053	0.103^*^	0.484^**^	0.098^*^	0.711^**^	0.225^**^	-

^*^p < 0.05, ^**^p < 0.01, ^***^p < 0.001. Lower left represents the value of Japanese non-religions, top right represents the value of Indonesian Muslims/Indonesian Christians.

### 4.2 Study 2. Relationship between depression recognition, causal attribution, and coping behavior

#### 4.2.1 Model fitness

In Study 2, we compared Indonesian Muslims, Japanese non-religions, and Indonesian Christians to examine the characteristics of the relationship among depression recognition, causal attribution, and coping behavior. The detail of the analysis was described in the Statictic Analysis section of Methods.

First, a multigroup analysis was used to examine the goodness-of-fit indices for the model without equality constraints for the three groups. The result yielded a χ^2^_(1, 404)_ = 3,021.280, *p* < 0.001, GFI = 0.839, AGFI = 0.807, RMSEA = 0.034, CFI = 0.874, AIC = 3,579.280, CMIN/DF = 2.152. Given the very large sample size of 995, we concluded that the significant χ^2^ value was unavoidable and may not imply that the model was inadequate. As all other goodness-of-fit indices were excellent, we judged that the fitness of the model was good. Therefore, configural invariance was guaranteed, and the factor structures of causal attribution and coping behavior developed in this study were adaptable to Indonesian Muslims, Japanese non-religions, and Indonesian Christians.

Then, we examined the differences in relationships among variables in the models between the three groups. Specifically, we compare the fitness of the model under two conditions: with and without equality constraints. The goodness of fit of the model without equality constraints was the same as above. However, the goodness of fit of the model with equality constraints on paths among the valuables of depression recognition, psychosocial attribution, biological attribution, religious attribution, help-seeking from professionals, religious coping, help-seeking from family and friends, and evil dispelling yielded a χ^2^_(1, 460)_ = 3,316.311, *p* < 0.001, GFI = 0.827, AGFI = 0.801, RMSEA = 0.036, CFI = 0.855, AIC = 3,762.311, CMIN/DF = 2.271. Hence, the goodness-of-fit of the model is acceptable.

Comparing the relative chi-square values (CMIN/DF) of the models with and without the equality constraint, the model without the equality constraint showed a lower value. In addition, the model without equality constraints was better for other goodness-of-fit indices. Specifically, the GFI, AGFI, and CFI were lower, and the RMSEA and AIC were higher in the model without equality constraints. Therefore, there were differences between the three groups.

**Figure 2:**
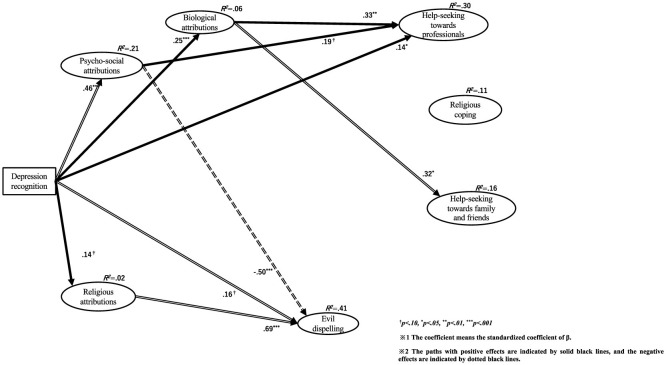
Relationships among depression recognition, causal attributions, and coping behaviors in Indonesian Muslims.

**Figure 3:**
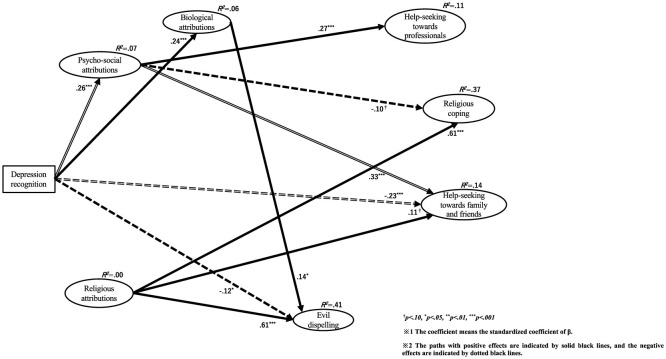
Relationships among depression recognition, causal attributions, and coping behaviors in Japanese non-religions.

**Figure 4:**
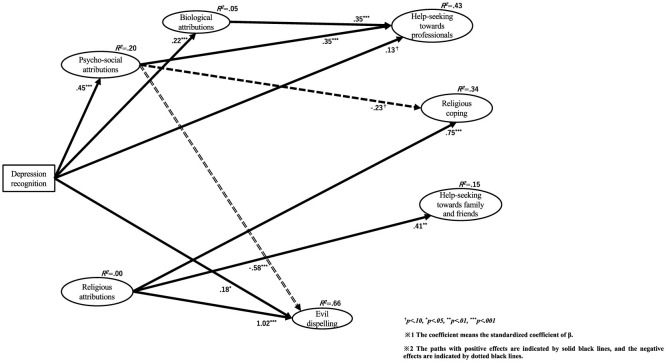
Relationships among depression recognition, causal attributions, and coping behaviors in Indonesian Christians.

As differences between the three groups were found, we used a pairwise comparison method to examine which groups differed from each other. To test the differences between the groups, the Bonferroni correction was used for the z-value, and the critical value was determined to be 2.42. The results are shown in Figure 1, and those for each z-value are summarized in Table 8. The Wald test results for each group are shown in the figures. The results of Indonesian Muslims, Japanese non-religions, and Indonesian Christians are shown in Figures 2–4, respectively.

**Table 8:** Standardized coefficients of paths in each group and results of pairwise comparisons.

			**1. Indonesian Muslims**		**2. Japanese non-religions**		**3. Indonesian Christians**		**Comparison between 1 and 2**	**Comparison between 1 and 3**	**Comparison between 2 and 3**
**Paths between variables**			**β**	***p***	**β**	***p***	**β**	***p***	**Statics (** ***z*** **)**		
Depresion recognition	→	Psycho-social attributions	0.461	0.000	0.260	0.000	0.451	0.000	**2.689**	−0.385	2.388
		Biological attributions	0.249	0.000	0.236	0.000	0.219	0.000	−0.092	−0.042	−0.133
		Religious attributions	0.143	0.079	−0.070	0.182	−0.041	0.573	2.205	−1.461	0.131
		Help-seeking toward professionals	0.144	0.040	0.012	0.805	0.133	0.067	1.539	−0.237	1.321
		Religious coping	0.003	0.971	−0.069	0.128	0.098	0.266	0.432	0.784	1.452
		Help-seeking toward family and friends	0.064	0.451	−0.231	0.000	0.045	0.618	**3.139**	−0.059	**2.533**
		Evil dispelling	0.160	0.078	−0.124	0.012	0.181	0.043	**3.054**	1.194	**3.089**
Psycho-social attributions	→	Help-seeking toward professionals	0.186	0.050	0.273	0.000	0.354	0.000	−0.909	1.156	0.405
		Religious coping	0.083	0.443	−0.101	0.093	−0.231	0.050	1.194	−1.97	−1.486
		Help-seeking toward family and friends	−0.175	0.132	0.331	0.000	−0.045	0.706	**−3.966**	0.589	**-2.598**
		Evil dispelling	−0.504	0.000	−0.096	0.142	−0.577	0.000	−0.739	**−2.846**	**−3.059**
Biological attributions	→	Help-seeking toward professionals	0.334	0.002	0.098	0.148	0.348	0.000	1.941	−0.367	1.867
		Religious coping	0.148	0.231	0.045	0.471	−0.123	0.306	1.012	**–**1.576	**–**1.174
		Help-seeking toward family and friends	0.323	0.015	−0.070	0.347	−0.011	0.927	**2.563**	**–**1.759	0.401
		Evil dispelling	0.119	0.399	0.139	0.044	−0.050	0.679	**–**1.137	−0.761	**–**1.526
Religious attributions	→	Help-seeking toward professionals	0.021	0.869	−0.049	0.381	−0.058	0.609	0.461	−0.378	0.109
		Religious coping	0.155	0.289	0.605	0.000	0.750	0.000	−0.585	1.686	2.07
		Help-seeking toward family and friends	0.182	0.241	0.109	0.077	0.413	0.005	0.445	0.719	1.656
		Evil dispelling	0.688	0.000	0.611	0.000	1.023	0.000	**-3.266**	**2.648**	−0.067

Bold type indicates that the value is above the critical value.

To avoid complications, the error terms are omitted from all figures, and the values of the partial correlations between the error terms are summarized in Table 9.

**Table 9:** Partial correlation between error terms in each group and results of pairwise comparisons.

			**1.Indonesian Muslims**		**2.Japanese non-religions**		**3.Indonesian Christians**		**Comparison between 1 and 2**	**Comparison between 1 and 3**	**Comparison between 2 and 3**
**Relevance of error terms**			***r***	***p***	***r***	***p***	***r***	***p***	**Statics (z)**		
e1 (Psycho-social attributions)	⇆	e2 (Biological attributions)	0.493	0.000	0.491	0.000	0.563	0.000	1.582	0.415	2.109
e1 (Psycho-social attributions)	⇆	e3 (Religious attributions)	0.525	0.000	0.197	0.000	0.604	0.000	**3.276**	1.836	**4.803**
e2 (Biological attributions)	⇆	e3 (Religious attributions)	0.525	0.000	0.119	0.058	0.678	0.000	**4.670**	2.319	**5.992**
e4 (Help-seeking toward professionals)	⇆	e5 (Religious coping)	0.356	0.000	0.142	0.016	0.349	0.000	**3.657**	−1.338	**2.843**
e4 (Help-seeking toward professionals)	⇆	e6 (Help-seeking toward family and friends)	0.194	0.026	0.476	0.000	0.342	0.000	−1.943	1.062	−0.762
e4 (Help-seeking toward professionals)	⇆	e7 (Evil dispelling)	0.227	0.038	0.171	0.010	0.203	0.098	−0.798	0.285	−0.382
e5 (Religious coping)	⇆	e6 (Help-seeking toward family and friends)	0.564	0.000	0.201	0.003	0.497	0.000	**4.613**	−0.611	**3.936**
e5 (Religious coping)	⇆	e7 (Evil dispelling)	0.554	0.000	0.596	0.000	0.248	0.113	0.665	−0.778	−0.441
e6 (Help-seeking toward family and friends)	⇆	e7 (Evil dispelling)	0.353	0.006	0.292	0.000	0.105	0.414	−1.427	−0.418	**–**1.342

Bold type indicates that the value is above the critical value.

#### 4.2.2 Path from depression recognition to 3 causal attributions

First, the path from depression recognition to psychosocial attribution showed a significant positive influence in all three groups (Indonesian Muslims β = 0.46, *p* < 0.001; Japanese non-religions β = 0.26, *p* < 0.001; Indonesian Christians = 0.45, *p* < 0.001). Pairwise comparisons revealed significant differences between Indonesian Muslims and Japanese non-religions.

Second, the path from depression recognition to biological attribution showed a significant positive influence on all three groups (Indonesian Muslims β = 0.25, *p* < 0.001; Japanese non-religions β = 0.24, *p* < 0.001; and Indonesian Christians β = 0.22, *p* < 0.001). Pairwise comparisons revealed no significant differences between groups.

Third, the path from depression recognition to religious attribution showed different effects in the three groups. Only Indonesian Muslims showed a marginally significant positive influence (β = 0.25, *p* = 0.079). However, in Japanese non-religions (β = −0.07, *p* = 0.182) and Indonesian Christians (β = −0.04, *p* = 0.573), there was no significant association but a negative association. Pairwise comparisons revealed no significant differences between groups.

The explanatory ratios for each causal attribution latent variable are as follows: Regarding psychosocial attributions, Indonesian Muslims was *R*^2^ = 0.21, Japanese non-religions was *R*^2^ = 0.07, and Indonesian Christians was *R*^2^ = 0.20. Relatively satisfactory explanatory rates were observed for Indonesian Muslims and Christians.

Regarding biological attributes, Indonesian Muslims was *R*^2^ = 0.06, Japanese non-religions was *R*^2^ = 0.06, and Indonesian Christians was *R*^2^ = 0.05. The explanation rates were inadequate for all three groups.

Regarding religious attributions, Indonesian Muslims was *R*^2^ = 0.02, Japanese non-religions was *R*^2^ = 0.00, and Indonesian Christians was *R*^2^ = 0.00. Specifically, for Japanese non-religions and Indonesian Christians, the explanation rates were below 0.00. The explanation rates were inadequate for all three groups.

#### 4.2.3 Path from depression recognition to 4 coping behaviors

First, the path from depression recognition to help-seeking from professionals showed a significant positive influence on Indonesian Muslims (β = 0.14, *p* = 0.040) and a marginally significant positive influence on Indonesian Christians (β = 0.13, *p* = 0.067). However, there was no association in Japanese non-religions (β = 0.01, *p* = 0.805). Pairwise comparisons revealed no significant differences between groups.

Second, the path from depression recognition to religious coping showed no significant effect in any of the three groups [Indonesian Muslims β = 0.00 (*p* = 0.971); Japanese non-religions β = −0.07 (*p* = 0.128); and Indonesian Christians β = 0.10 (*p* = 0.266)]. Pairwise comparisons revealed no significant differences between groups.

Third, the path from depression recognition to help-seeking from family and friends showed a significant negative influence on Japanese non-religions (β = −0.23, *p* < 0.001). However, there was no significant effect on Indonesian Muslims (β = 0.06, *p* = 0.451) and Indonesian Christians (β = 0.05, *p* = 0.618). Pairwise comparisons showed significant differences between Indonesian Muslims and Japanese non-religions and between Indonesian Christians and Japanese non-religions.

Fourth, the path from depression recognition to evil dispelling showed different effects among the three groups. In Indonesian Muslims (β = 0.16, *p* = 0.078) and Indonesian Christians (β = 0.18, *p* = 0.043), there was a marginally significant and significant positive effect, respectively. Alternatively, in Japanese non-religions, there was a significant negative effect (β = −0.12, *p* = 0.012). Pairwise comparisons showed significant differences between Indonesian Muslims and Japanese non-religions and between Indonesian Christians and Japanese non-religions.

#### 4.2.4 Path from psychosocial attribution to 4 coping behaviors

First, the path from psychosocial attribution to help-seeking from professionals showed a marginally significant or significant positive influence in all three groups. Specifically, it was Indonesian Muslims β = 0.19 (*p* = 0.050), Japanese non-religions β = 0.27 (*p* < 0.001), and Indonesian Christians β = 0.35 (*p* < 0.001). Pairwise comparisons revealed no significant differences between groups.

Second, the path from psychosocial attribution to religious coping showed different effects in the three groups. In Indonesian Muslims, there was no significant association (β = 0.08, *p* = 0.443), but there was a marginally significant negative influence on Japanese non-religions (β = −0.10, *p* = 0.093) and a significant negative influence on Indonesian Christians (β = −0.23, *p* = 0.050). Pairwise comparisons revealed no significant differences between groups.

Third, the path from psychosocial attribution to help-seeking from family and friends showed no significant association in Indonesian Muslims (β = −0.18, *p* = 0.132) and Indonesian Christians (β= −0.05, *p* = 0.706). However, in Japanese non-religions, there was a significant positive influence (β = 0.33, *p* < 0.001). Pairwise comparisons showed significant differences between Indonesian Muslims and Japanese non-religions and between Indonesian Christians and Japanese non-religions.

Fourth, the path from psychosocial attribution to evil dispelling showed a significant negative influence on Indonesian Muslims (β = −0.50, *p* < 0.001) and Indonesian Christians (β = −0.58, *p* < 0.001). However, there was no significant association in Japanese non-religions (β = −0.10, *p* = 0.142). Pairwise comparisons showed significant differences between Indonesian Muslims and Indonesian Christians and between Indonesian Christians and Japanese non-religions.

#### 4.2.5 Path from biological attribution to 4 coping behaviors

First, the path from biological attribution to help-seeking from professionals showed a significant positive influence on Indonesian Muslims (β = 0.33, *p* < 0.001) and Indonesian Christians (β = 0.35, *p* < 0.001). However, there was no association in Japanese non-religions (β = 0.10, *p* = 0.148). Pairwise comparisons revealed no significant differences between groups.

Second, the path from biological attribution to religious coping showed no significant association in any group. Indonesian Muslims was β = 0.15 (*p* = 0.231), Japanese non-religions were β = 0.05 (*p* = 0.471) and Indonesian Christians was β = −0.12 (*p* = 0.306). Pairwise comparisons revealed no significant differences between groups.

Third, the path from biological attribution to help-seeking from family and friends showed significant positive influence only in Indonesian Muslims (β = 0.32, *p* = 0.015). Alternatively, there was no significant effect on Japanese non-religions (β = −0.07, *p* = 0.347) and Indonesian Christians (β = −0.01, *p* = 0.927). Pairwise comparisons revealed significant differences between Indonesian Muslims and Japanese non-religions.

Fourth, the path from biological attribution to evil dispelling showed significant positive influence only in Japanese non-religions (β = 0.14, *p* = 0.044). However, there was no significant association between Indonesian Muslims (β = 0.12, *p* = 0.399) and Indonesian Christians (β = −0.05, *p* = 0.679). Pairwise comparisons revealed no significant differences between groups.

#### 4.2.6 Path from religious attribution to 4 coping behaviors

First, the path from religious attribution to help-seeking from professionals showed no significant association in any of the three groups. Indonesian Muslims was β = 0.02 (*p* = 0.869), Japanese non-religions were β = −0.05 (*p* = 0.381) and Indonesian Christians was β = −0.06 (*p* = 0.609). Pairwise comparisons revealed no significant differences between groups.

Second, the path from religious attribution to religious coping showed no significant association only in Indonesian Muslims (β = 0.16, *p* = 0.289). Alternatively, there was a significant positive influence on Japanese non-religions (β = 0.61, *p* < 0.001) and Indonesian Christians (β = 0.75, *p* < 0.001). Pairwise comparisons revealed no significant differences between groups.

Third, the path from religious attribution to help-seeking from family and friends showed no significant association only in Indonesian Muslims (β = 0.18, *p* = 0.241). However, there was a marginally significant positive influence on Japanese non-religions (β = 0.11, *p* = 0.077) and a significant positive influence on Indonesian Christians (β = 0.41, *p* < 0.001). Pairwise comparisons revealed significant differences between Indonesian Muslims and Japanese non-religions.

Fourth, the path from religious attribution to evil dispelling showed a significant positive influence in all three groups. Specifically, it was Indonesian Muslims β = 0.69 (*p* < 0.001), Japanese non-religions β = 0.61 (*p* < 0.001), and Indonesian Christians was β = 1.02 (*p* < 0.001). Pairwise comparisons showed significant differences between Indonesian Muslims and Japanese non-religions and between Indonesian Muslims and Indonesian Christians.

The explanatory ratios for each coping behavior latent variable were as follows: In help-seeking from professionals, Indonesian Muslims was *R*^2^ = 0.30, Japanese non-religions was *R*^2^ = 0.11, and Indonesian Christians was *R*^2^ = 0.43. Satisfactory explanatory rates were observed, particularly for Indonesian Muslims and Christians.

Regarding religious coping, Indonesian Muslims was *R*^2^ = 0.11, Japanese non-religions was *R*^2^ = 0.37, and Indonesian Christians was *R*^2^ = 0.34. Satisfactory explanatory rates were observed, especially for Japanese non-religions and Indonesian Christians.

Regarding help-seeking from family and friends, Indonesian Muslims was *R*^2^ = 0.16, Japanese non-religions was *R*^2^ = 0.14, and Indonesian Christians was *R*^2^ = 0.15. Relatively satisfactory explanatory rates were observed for all three groups.

For evil dispelling, Indonesian Muslims was *R*^2^ = 0.41, Japanese non-religions was *R*^2^ = 0.41, and Indonesian Christians was *R*^2^ = 0.66. Satisfactory explanatory rates were observed in all three groups.

## 5 Discussion

This study aimed to use quantitative methods to clarify the relationship between depression recognition, causal attribution, and coping behavior in Indonesian Muslims. Specifically, we investigated the characteristics of the relationship of each variable with religious coping. As mentioned earlier, we included university students from both countries for several reasons: they are a high-risk group for mental health problems because they are adolescents and have low adaptation to the host culture; MHL acquired at this age influences their later life; and we wanted to control for the effects of education. Interestingly, even among university students whose knowledge and beliefs are influenced by Westernized academia, the results still reflect cultural differences. Below, we discuss the characteristics of the mechanisms leading to each of the four coping behaviors among Indonesian Muslims: help-seeking from professionals, religious coping, help-seeking from family and friends, and evil dispelling.

### 5.1 Mechanisms leading to help-seeking from mental health professionals

The relationship between depression recognition and causal attribution with help-seeking from (mental health) professionals among Indonesian Muslims is as follows. Specifically, depression recognition had a significant positive effect on psychosocial and biological attribution. Depression recognition, psychosocial attribution, and biological attribution all had a significant positive effect on help-seeking from professionals.

It has been pointed out in a former MHL study that being able to identify mental illness based on the symptoms could lead to help-seeking from professionals (Yap et al., 2015). This is what has been said mainly in studies conducted in Western countries; however, the same result has also been suggested in Indonesia. In psychiatric patient assessment, assessing the patient's condition based on biological, psychological, and social factors using a bio-psychosocial model is recommended (Setyonegoro, 1965; Shimoyama, 2010). Hence, as previous studies on MHL have suggested, an increase in knowledge, understanding, or recognition of Western medical correctness could promote help-seeking from mental health professionals. Therefore, the results of this study with college students align with the findings of previous MHL studies conducted on various age groups.

### 5.2 Mechanisms leading to religious coping

Regarding religious coping among Indonesian Muslims, no significant associations were found with any of the variables addressed in this study. However, the mean value of religious coping in Indonesian Muslims was 2.14 (*SD* = 0.070, max = 4), which was never lower than help-seeking from professionals 2.43 (*SD* = 1.07, max = 4) or help-seeking from family and friends 2.17 (*SD* = 0.073, max = 4). Furthermore, there was a significantly strong positive correlation between religious coping and religiosity (*r* = 0.71, *p* < 0.001).

According to Kosugi (2009), there are five obligatory acts called “the five pillars of Islam,” and one of them is to pray five times a day facing the direction of the Qibla (Mecca). The six subscales of “religious coping” in this study are all inferred to have been related to these pillars. Therefore, it was considered that religious coping in this study is not an action performed in relation to mental illness recognition, knowledge, or attribution of symptoms of depression but an action that is routinely engaged in the Islamic faith. Further investigations are warranted to determine whether similar findings can be obtained.

### 5.3 Mechanisms leading to evil dispelling

Regarding evil dispelling among Indonesian Muslims, depression recognition had a marginally significant positive effect, while religious attribution had a significant positive impact. However, though psychosocial attribution had a significant negative influence, biological attribution had no association. In Indonesian Christians, the results were similar, but depression recognition was not associated with religious attribution. In addition, the only significant correlation between depression recognition and evil dispelling in Indonesian Muslims was negative (*r* = −0.13, *p* = 0.037). Additionally, among Japanese non-religions, similar to Indonesian Christians, depression recognition was not significantly associated with religious attribution; religious coping had a significant positive effect on evil dispelling, whereas depression recognition had a significant negative effect on evil dispelling. The results of the between-group comparison showed that biological attribution had a significant positive effect on evil dispelling among Japanese non-religions, although no significant differences were found between the groups.

In Indonesian Islamic culture, there is a ceremony called *ruqyah* to expel spirits. According to a survey of Islamic leaders in Indonesia (ustadz; Araki, 2022), *ruqyah* refers to the act or treatment of expelling spirits according to the teachings of Islam for those who are possessed by bad spirits such as *jinn* or *Satan*, which interfere with their lives or cause them to feel physically or mentally unwell. Specifically, for those who have mental or physical ailments, ustadz touch the person's body while reciting the Qur'an chapters to expel *jinn* or Satan; in many cases, *jinn* or Satan is expelled from the mouth of the person who is ailing. In addition, it has been reported that in this process, Qur'an chapters are chanted while spraying water in a cup, and then water [*air doa*: water with prayer] is given to the person to drink (Araki, 2022).

The subscales of “evil dispelling” in this study consisted of three items, “buy an amulet or talisman,” “have a purification performed,” or “use the purified water (drink it or bathe in it).” As these processes are also used in *ruqyah*—for example, by drinking water purified by the Qur'an chapters—it can be inferred that evil dispelling among Indonesian Muslims in this study is equivalent to *ruqyah*.

In addition, those who wish to receive *ruqyah* are generally those who have physical or mental problems, which in some cases can be cured by visiting a Western medical doctor, but in the Islamic context, many of the causes of such things can be possession by evil spirits, such as *jinn* or Satan (Araki, 2022). In Ohtsuka (2002), *jinn* are supernatural beings, such as spirits, recognized in Islam and feared as beings that possess people and torment them with diseases and calamities. This leads us to believe that in Indonesian Islamic culture, possession by evil spirits is a possible cause of illness.

The subscales of “religious attribution” include items such as “be possessed by something,” “be a curse upon one's ancestors,” and “be put under black magic,” that is, be adversely affected by supernatural beings. Additionally, depression recognition had a marginally significant positive effect on religious attribution. Although it remained mariginally significant, it was inferred that the results of this study reflect the possibility of considering religious factors, such as possession by evil spirits, in mental illness in Indonesian Islamic culture.

By contrast, among Indonesian Christians, there was no association between depression recognition and religious attribution. Thus, the results suggest that Indonesian Christians may use coping behaviors and dispelling to reduce depression symptoms when their religious attribution is unrelated to the recognition of depression. In Christians, particularly Catholics, demon dispelling, that is, exorcism, is officially approved (Christian Today, 2014). In terms of dispelling evil, both Christian exorcism and Islamic *ruqyah* are similar. However, Christians, particularly Catholics, are said to engage in exorcism only if a medical doctor confirms that they do not have a mental or physical illness (Wilkinson, 2007). This is consistent with the results of Indonesian Christians in this study. Thus, the use of evil dispelling for depression symptoms may be caused by different mechanisms between Indonesian Christians and Muslims.

However, there were no significant differences among the three groups regarding the relationship between recognition of depression and religious attribution. Therefore, it is necessary to study the differences with a larger sample size and improved translation and measurement methods to verify this study's findings.

### 5.4 Mechanisms leading to help-seeking from family and friends

In Indonesian Muslims, biological attribution has a significant positive effect on help-seeking from family and friends. A three-group comparison revealed a significant difference between Indonesian Muslims and non-religious Japanese but no difference between Indonesian Muslims and Christians. In Indonesian Christians, biological attribution was not associated with help-seeking from family or friends. The hadiths, the second set of Islamic books, include the sayings and deeds (sunnah) of the prophet Muhammad (peace be upon him) that Muslims observe in their religious lives (Kosugi, 2009).

Abu Hurairah heard the Messenger of God say: “There are five obligations that a Muslim should perform toward a Muslim: to respond to greetings, to visit the sick, to attend funerals, to accept invitations, and to wish good luck to a person who sneezed” (Translated, Makino, 1993).

What is stated as a commentary on this second obligation, “visiting the sick,” is that when a Muslim becomes ill, and they are alone at home or in a hospital, it is obligatory for one of their fellow Muslims to visit them for sympathy, that is, an obligation in the Muslim community (Hadith Encyclopedia, 2023). Hence, in the Muslim context, it can be inferred that a sick person (in the Western medical context) has the right and obligation to be visited by someone who is also Muslim. However, to the best of our knowledge, Christianity does not explicitly state such rights and obligations. Therefore, it was hypothesized that biological attribution would have a positive effect on seeking help from family and friends among Indonesian Muslims. However, further research is needed as this study did not find significant differences between Indonesian Muslims and Christians.

Additionally, the region of Indonesia targeted in this study, near Jakarta, falls within the Java category. Given that this study was conducted among college students, the following points may be considered. In Javanese culture, there is an ideal person image, and the parent–child relationship is not considered equal, and parents have overwhelming decision-making power over their children's lives during adolescence and young adulthood to develop their children into ideal persons (Hasanah et al., 2019). Another study found that one of the factors contributing to depression among young Indonesians is the lack of equal parent–child relationships and poor family relationships, such as parents being coercive toward their children (Brooks et al., 2022b).

This suggests that in Indonesia, there was no association between psychosocial attribution and help-seeking from family and friends because of the possibility of being blamed by parents and family members for their illness when attributing depression to psychosocial factors such as “lack of achievement” or “major failure.” This aspect needs further investigation. It is necessary to examine whether similar findings can be obtained when variables related to parent–child relationships are used.

## 6 Conclusion

This study targets university students for the reasons mentioned above, and it is not only meaningful for university students to consider how to provide support focused on their results but also seems to be somewhat generalizable to other age groups, especially those in adolescence and beyond.

The results of this study suggest two points regarding appropriate future Japanese mental health services for Indonesian Muslims. The first point concerns MHL improvement, which is one of the objectives of MHL research, which is to promote help-seeking from mental health professionals. However, there is an ongoing debate on what kind of psychoeducation would promote MHL (Nakamura, 2021).

To the best of our knowledge, most of the previous MHL studies in Japan have been conducted with Japanese participants, and the content of psychoeducation has been developed based on these results (ex. Ojio et al., 2016).

However, this study suggests that the process, from the level of recognition of mental illness and understanding of causal attribution to the selection of coping behaviors, may differ according to culture, religion, and country. In other words, it is possible that different recognitions and understandings underlie seemingly identical coping behaviors, depending on individual and group differences. This is consistent with what has been said in medical anthropology that the understanding of illness and therapeutic coping differs across cultures (Kleinman, 1988, translated by Eguchi et al., 2015). Therefore, when developing MHL psychoeducation for Indonesian Muslims in Japan, it is necessary to consider modifying the content for Indonesian Muslims by adding a religious context for mental illness and disease to the Japanese content.

Furthermore, it is important to consider the implications of this study for providing therapeutic support. For example, a case study in Malaysia reported the effectiveness of a religious coping ritual, *ruqyah*, in conjunction with Western medical treatment for a Muslim patient who developed depression (Razali et al., 2018). Hence, in therapeutic support, mental health professionals need to be ready to combine both Western medicine and Islamic religious coping as required by their patients. Specifically, the need for collaboration between mental health professionals and religious leaders has been reported, considering patient gains (Hasan and Tanjung, 2017), and such efforts should be considered for future mental health services in Japan. It is important to develop specific discussions regarding this.

The results also suggest the need for mental health professionals in Japan to understand the cultural diversity of MHL. Since the 1970s, clinical bias, or the distortion of clinical judgments and attitudes caused by the stereotypical views held by therapists toward certain groups in terms of race, age, or gender, has been reported in the West (Wisch and Mahalik, 1999). Such bias may also influence the therapeutic relationship between Japanese non-religion therapists and Indonesian Muslim patients.

An example of possible bias in this study is that Indonesian Muslims do not engage in religious coping or religious attribution for depression because they cannot recognize it as a mental illness. However, stereotypical views can mislead Japanese mental health professionals to believe that such attribution or coping is wrong and needs to be modified.

If such misunderstandings arise among Japanese therapists, these misunderstandings may become a barrier to providing support. Indeed, in international literature, differences in religious beliefs between patients and professionals have been noted as a barrier to help-seeking behaviors (Abe-Kim et al., 2004). Therefore, as suggested in this study, it would be beneficial to share with or educate Japanese professionals on the differences in their beliefs regarding mental illnesses.

In addition, it is necessary to identify beliefs regarding mental illnesses that are specific to diverse cultural minorities. Mental health professionals need to understand the influence of cultural diversity on beliefs regarding mental illness and reduce the possible disadvantages to patients and clients from different cultures by providing culture-appropriate treatment.

While the findings above are significant, it is important to discuss future perspectives as well. First, for some of the factors in the scales used in this study, α was not as high or the value of the explanatory coefficient for SEM was not as high as expected. This in itself does not overturn the SEM results obtained in this study, and the results of this study are informative in themselves. However, it is possibility that the scales used in this study did not fully capture the subtle differences in languages and cultures across th countries. It is complicated to develop a single scale that can measure phenomena and matters that differ greatly among cultures; however, development of such improved scales is expected in the future.

In addition, while we focuced in the participants in this study for the reasons mentioned earlier, future research on subjects not examined in this study is needed. For example, in pursuing the diversity of mental health services, it will be important to study subjects with other cultural backgrounds, such as other Indonesian religious groups, who were not included in this study. Moreover, while this study targeted university students for research purposes, it would be meaningful to examine the detailed differences in characteristics between university students and other age groups in the future. Furthermore, to examine the quantitative relationships among the targeted variables, this study targeted individuals with various possible MHLs. Conducting quantitative research specifically focused on individuals with depression would be beneficial.

## Data Availability

The raw data supporting the conclusions of this article will be made available by the authors, without undue reservation.
